# Human single-stranded DNA binding proteins are essential for maintaining genomic stability

**DOI:** 10.1186/1471-2199-14-9

**Published:** 2013-04-01

**Authors:** Nicholas W Ashton, Emma Bolderson, Liza Cubeddu, Kenneth J O’Byrne, Derek J Richard

**Affiliations:** 1 Genome Stability Laboratory, Cancer and Ageing Research Program, Institute of Health and Biomedical Innovation, Translational Research Institute, Queensland University of Technology, Woolloongabba, Queensland, 4102, Australia; 2 School of Science and Health, University of Western Sydney, Sydney, Locked Bag 1797, Penrith, NSW, 2751, Australia

**Keywords:** Single-stranded DNA binding proteins (SSBs), Oligonucleotide/oligosaccharide binding (OB)-fold, Double-strand DNA break (DSB) repair, Homology-directed repair (HDR), Translesion synthesis, Nucleotide excision repair (NER), Replication fork restart, Cell cycle checkpoint activation, Telomere maintenance

## Abstract

The double-stranded conformation of cellular DNA is a central aspect of DNA stabilisation and protection. The helix preserves the genetic code against chemical and enzymatic degradation, metabolic activation, and formation of secondary structures. However, there are various instances where single-stranded DNA is exposed, such as during replication or transcription, in the synthesis of chromosome ends, and following DNA damage. In these instances, single-stranded DNA binding proteins are essential for the sequestration and processing of single-stranded DNA. In order to bind single-stranded DNA, these proteins utilise a characteristic and evolutionary conserved single-stranded DNA-binding domain, the oligonucleotide/oligosaccharide-binding (OB)-fold. In the current review we discuss a subset of these proteins involved in the direct maintenance of genomic stability, an important cellular process in the conservation of cellular viability and prevention of malignant transformation. We discuss the central roles of single-stranded DNA binding proteins from the OB-fold domain family in DNA replication, the restart of stalled replication forks, DNA damage repair, cell cycle-checkpoint activation, and telomere maintenance.

## Introduction

DNA exists primarily as a duplex to stabilise and protect our genome. However, as a result of many cellular processes, such as replication and transcription, single-stranded DNA (ssDNA) is exposed. While a necessary metabolic intermediate, these exposed stretches are vulnerable to both chemical and enzymatic degradation, and as such must be sequestered. In this process, the single-stranded DNA binding protein family (SSBs) are essential cellular components [1-4]. In addition to this role, SSBs function in the correct processing of ssDNA, including the recruitment of appropriate functional enzymes. In the current review, we discuss the roles of a subset of human SSBs in the maintenance of genomic stability, an essential consideration in the prevention of malignant transformation and loss of cellular viability.

**Figure 1:**
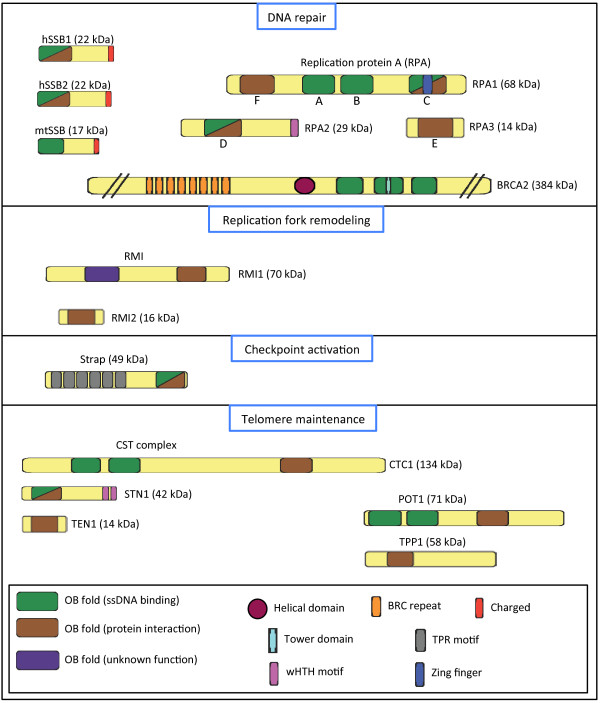
**Human OB-fold containing proteins are essential for multiple aspects of genome stability maintenance.** Schematic representation of human OB-fold containing proteins, illustrating their predicted domains and central function. Domains are not drawn to scale.

The characteristic functional unit of the SSBs is the oligonucleotide/oligosaccharide-binding (OB)-fold, a protein domain that facilitates binding to ssDNA, as well as various protein-protein interactions (Figure 1). As we have described previously [1], the SSB family consists of two core sub-groups; the simple SSBs, which contain one OB-fold per polypeptide, and the higher order SSBs, which contain multiple OB-folds (which may be on different polypeptides). The human genome encodes both simple and higher order SSBs: the simple SSBs are represented by human single-stranded DNA binding proteins 1 and 2 (hSSB1 and 2) and the mitochondrial SSB (mtSSB), while higher order SSBs are represented by heterotrimeric RPA. In addition, other proteins have also adopted the ssDNA-binding-OB-fold structure within their polypeptides and may be considered members of the SSB family. For instance the serine/threonine kinase receptor associated protein (Strap) structurally contains one DNA binding OB fold as do the simple SSBs, while the TPP1 - protection of telomeres 1 (POT1) breast cancer 2, early onset (BRCA2) and the CST complex form complexes reminiscent of higher order SSBs. While containing only a single OB-fold, the majority of simple SSBs, including all human simple SSBs, do however assemble as higher order multiple OB-fold containing oligomers [5-7]. This is exemplified by hSSB1, which is predominantly dimeric in solution and may shift to a stable tetrameric conformation following activation by the ataxia telangiectasia mutated kinase (ATM) [unpublished data from within our group].

### DNA binding

The ssDNA-binding characteristic of the SSBs is largely facilitated by the OB-fold [4,8]. This domain comprises five antiparallel β-strands arranged as a cylindrical β-barrel, capped at one end by an α-helix between the third and fourth β-strands [8]. OB-folds interact with their ssDNA substrates through nucleotide base stacking with aromatic residues in the binding cleft and electrostatic interactions with the phosphodiester backbone [9-20]. While ssDNA-binding of the OB-fold is in most cases largely non-specific, OB-fold mediated DNA binding of the telomere associated protein POT1 is seen to be highly specific for telomeric repeats [21]. This may be due to the high pyrimidine content of the telomeric single strand where POT1 binds. Thermodynamic studies of *Escherichia coli* SSB-ssDNA interactions show that purines (due to the double-ring) may not sterically fit into the SSB ssDNA-binding cleft compared to pyrimidines [22,23]; a preference for binding to pyrimidine-rich sequences has also been noted for hSSB1 [7]. Telomere specific binding is also observed for the STN1 protein, however this seems to be due to two C-terminal winged helix-turn-helix domains, rather than specificity of the OB-fold [24].

Eukaryotic RPA is a heterotrimeric complex composed of ~70 kDa, ~32 kDa, and ~14 kDa subunits (RPA1, RPA2 and RPA3, respectively) (Figure 1). Between them, the subunits contain 6 OB-folds, which are homologous to those of the simple SSBs; these are designated A-F, in order of their respective DNA-binding affinities. Of the OB-folds in the RPA complex, four bind ssDNA. To date, two RPA DNA binding conformations have been designated [25,26]. In one, facilitated by the A and B OB-folds of RPA1, RPA binds ssDNA with low-affinity, occluding a region of ~8 nucleotides (nt) [27,28]. The additional contribution of the C and D OB-folds then allows RPA to bind ssDNA with a high-affinity, where ~30 nt are occluded [29]. Associated with these discrete binding modes is a difference in protein-protein interactions; while the A-OB-fold interacts with various proteins in the low-affinity mode, there is a considerable decrease following transition to the high-affinity state [30]. Furthermore, in the high-affinity state, RPA binds ssDNA with a 5^′^ to 3^′^ polarity, whereby RPA1 is positioned 5^′^ to RPA2; this arrangement has a considerable effect on the functionality of the RPA complex [26,31-33].

The recently identified hSSB1 and 2 exemplify simple SSBs in the human genome [7]. Both proteins are structurally similar, possessing a single N-terminal OB-fold, as well as a basic C-terminus [1]. For hSSB1, the C-terminus is involved in a protein-protein interaction with NBS1, a component of the MRE11-NBS1-RAD50 (MRN) repair complex [34]. Agarose gel shift analysis using virion phiX174 ssDNA as a substrate has indicated that the hSSB1 dimer occludes a region of ~12 nt (5–6 nt per monomer) [1]. As yet, DNA binding activity has not been described for hSSB2. As well as binding DNA, the OB-fold of hSSB1 (and presumably hSSB2) has been shown to interact with the integrator complex subunit 3 (INTS3) [35].

Recent data has suggested the interaction with INTS3 leads to diminished hSSB1 ssDNA-binding, as demonstrated by an increase in observed K_d_ for a ssDNA substrate (15 nm [36] to 45 nm [34]) when in complex. Interestingly, a similar ssDNA-binding affinity has been observed between unbound hSSB1 and the RPA heterotrimer [36]. While both RPA and hSSB1 show high specificity for ssDNA, hSSB1 has also recently been shown to bind short (33 nt) duplex DNA constructs, as well as duplex DNA with short 6 bp overhangs [37]. In this instance, hSSB1 was suggested to bind ssDNA at natural breathing sites in the constructs rather than binding the dsDNA. As a number of SSBs are capable of melting dsDNA, such as the SSB from *Sulfolobus solfataricus*[38], this may suggest a similar activity for hSSB1. This is consistent with data that show the minimal ssDNA length required for binding of hSSB1 is reduced when it is adjacent to duplex DNA [36]. Interestingly, unlike the bacterial SSBs, which wrap 65 nt of DNA around their tetrameric structure [39], both RPA and hSSB1 bind ssDNA in an extended fashion, presumably to allow access of other DNA interacting factors [1,36,40].

### SSBs are required for DNA replication

The accurate replication of DNA is a central process in the maintenance of a stable genome. For this, RPA has a central role in both replication initiation and progression. Despite the central importance of this process, RPA appears to function as the sole required SSB, and neither hSSB1 nor 2 have been found to co-localise with replication foci or to influence S-phase progression [7,37]. In the earliest stage of eukaryotic DNA replication, sites of initiation are bound by origin recognition complexes (ORC), which stimulate the accumulation of downstream replicative factors. These include Cdc6 and Cdt1, both of which are required for the loading of minichromosome maintenance proteins (MCMs), which form the pre-initiation complex [41].

Following MCM mediated ssDNA exposure, RPA binds these stretches, where the 70 kDa subunit interacts with and stabilises the DNA polymerase α-primase complex (Pol α) [42,43]. Pol α is required for the synthesis of short oligonucleotide primers from which the more processive DNA polymerases δ and ε (Pol δ and ε) can synthesise the new lagging and leading strands, respectively [44]. Therefore, Pol α is required for initiating leading strand synthesis, as well as for the production of the ~ 20 million Okazaki fragment primers [45]. Here the primase subunit of Pol α firstly synthesises an RNA strand of 7 – 12 nt, which is extended with an additional short DNA chain (~ 20 nt) by the polymerase subunit [46,47]. The efficiency of Pol α chain extension appears reliant on RPA, where it has been reported to act as a enhancer of polymerase processivity and fidelity [48].

To allow access of Pol δ and ε, the Pol α complex must be removed following primer synthesis. This is at least partially facilitated by replication factor C (RFC), which competitively binds RPA, and disrupts its interaction with Pol α [42]. In addition, RFC is an essential clamp loader of the proliferating cell nuclear antigen (PCNA) sliding clamp [49]. PCNA is a central protein in the initiation and persistence of DNA replication, where it functions as a processivity factor for DNA polymerase δ [50]. PCNA encircles the duplex DNA by virtue of its ring-like structure, formed by three PCNA monomers; the inner surface of the homotrimeric clamp is rich in basic residues, allowing interaction with the DNA phosphodiester backbone, while the outer surface of PCNA interacts with various replication factors, thereby tethering them to sites of replication [51,52]. The absolute requirement of RPA and PCNA in mediating primer extension has lead to the co-localisation of these proteins being widely used as a marker of replication [53,54].

The discontinuous synthesis of the lagging strand means that each section will eventually collide with the initiating primer of the downstream fragment; this process results in displacement of short oligonucleotide flaps, which must be removed. In eukaryotic cells, this is facilitated by two major pathways, depending on the length of the displaced section [55]. In the first, short displaced ssDNA fragments of 3–5 nt are recognised and cleaved by the flap endonuclease (FEN1), leaving behind a nick in the phosphodiester backbone, which is sealed by DNA ligase [56-58]. *In vitro* data have however suggested that while the majority of these flaps are removed by FEN1, a small portion are missed, such that they form lengthier regions [59,60]. In this instance, the second pathway seems to be initiated where the exposed ssDNA is bound by RPA [61,62]. This event appears necessary for the recruitment of the Dna2 helicase/nuclease, which cleaves the flap and generates shorter 5–7 nt overhangs to be processed by FEN1 [61,62].

The replicative activity of RPA is partially governed by phosphorylation events. During S and G2/M of the cell cycle, the N-terminus of RPA2 is phosphorylated at S23 by cyclin dependent kinase 2 (CDK2)-cyclin B. The functional consequence of this modification is however unclear, and contradictory findings regarding the role of this event have been reported. Indeed, while addition of purified CDK2-cyclin B has been shown to stimulate replication *in vitro*[63], S23 mutated RPA2 is still able to support DNA synthesis [64,65]. At the initiation of mitosis, S29 of RPA2 is also phosphorylated by CDK2 [66]. This modification appears to deactivate RPA, as the DNA binding affinity of the complex purified from mitotic cells appears reduced when compared to the de-phosphorylated form [67]. This is supported by the finding that purified CDK2 is able to disassemble RPA foci from interphase chromatin [68]. Phosphorylation of RPA2 has also been demonstrated following DNA damage induction as a means of inhibiting RPA replicative activity [69-71]. This was observed after both oxidative [71] and replication-mediated damage [72] during S-phase, as well as throughout the cell cycle as a result of double-strand DNA break formation [73]. In these cases, damage activates the phosphatidylinositol 3-kinase related kinases: ATM, ATM and Rad3 related kinase (ATR) and DNA-dependent protein kinase (DNA-PK); each of these kinases phosphorylate RPA2 at five N-terminal residues [73-82]. Following DNA damage repair, or migration out of M phase, RPA2 must then be de-phosphorylated to reset replicative potential [83,84]. This has been suggested to occur via the 1A/2A protein phosphatases (PP1A and PP2A, respectively), as RPA de-phosphorylation is suppressed following inhibition of these enzymes using okadaic acid [84]. This has been further supported by RNA interference data, which has indicated a role for PP2A in de-phosphorylation of the RPA T21 and S33 ATM/ATR phosphorylated residues [85].

### SSBs are essential for the stabilisation and restart of stalled replication forks

As mentioned above, RPA is essential for the replication of DNA during S-phase, functioning both in the establishment and elongation of the replicative fork [1]. In the event of DNA damage, these processes may however be interrupted, causing the fork to stall [86]. Here SSBs also play an important role both in the stabilisation and protection of exposed ssDNA, as well as in re-initiation of fork migration. Additionally, the collapse or disassembly of the replicative machinery may result in the formation of single- or double-stranded DNA breaks, the repair of which requires the concerted effort of SSBs. In a typical scenario, replication fork migration is disrupted by a bulky lesion, which inhibits polymerase activity. Alternatively, certain regions of genetic material are apparently more difficult to replicate, and as such are subject to high rates of fork stalling. These sites include so-called ‘fragile sites’, regions of the human genome associated with increased genetic instability [87].

Following fork stalling, repair of the damaged region and restart of replication may be facilitated through two main mechanisms: homology directed repair (HDR) and translesion synthesis (TLS). While HDR makes use of a homologous region found in an opposing strand, TLS instead utilises a set of low-fidelity polymerases to replicate through the damage site, potentially introducing base mismatches [88].

#### Homology directed repair

One of the most immediate requirements in the prevention of stalled replication fork collapse is the stabilisation of exposed ssDNA; this is in part facilitated by the rapid accumulation of RPA [89,90]. Here, the requirement of ssDNA-binding is further amplified by helicase uncoupling from the replicative machinery [91,92]. In this role, RPA functions both to protect the exposed ssDNA, as well as to interact with a number of proteins involved in both stabilising and remodelling the replication fork. An example of this is the localisation of RPA with the MRN complex at sites of replicative stress [90,93]. Here the RPA1 N-terminal OB-fold has also been shown to interact directly with Mre11, an association required for S-phase checkpoint activation [94-96]. In addition, NBS1 of the MRN complex appears necessary for hyper-phosphorylation of the 32 kDa RPA subunit [93], a modification mediated by the central DNA repair kinase ATR [97,98]. Recently hyper-phosphorylation of RPA has been shown as stimulatory for RAD51 foci formation at replication forks stalled by hydroxyurea treatment [99]. The formation of RAD51 nucleofilaments is an essential aspect of homology directed strand invasion. Stabilisation of the RAD51 coated strand is also promoted by an interaction between RAD51 and the C-terminus of BRCA2, an interaction that is dispensable for the repair of double-strand DNA breaks but appears important in the prevention of fork collapse [100].

The RPA2 subunit is also known to interact directly with Timeless-interacting protein (Tipin) [101,102]. Tipin is a component of the replication pausing complex (RPC), a protein complex that is involved in the coordinated pausing of both polymerase and helicase migration at stalled replication forks [103-112]. Following replication fork disruption, RPA stabilises the Timeless (Tim1)-Tipin heterodimer, a central unit of the RPC, at sites of DNA damage [102]. Furthermore, this interaction appears to promote the stabilisation of claspin, a Tipin binding partner required for efficient S-phase checkpoint activation [102]. In agreement with these findings, Tim1-Tipin depleted cells show deficient intra-S checkpoint activation, including deficient Chk1 phosphorylation [101,112-114].

A central aspect of replication restart is remodelling of the stalled replicative fork; this is largely facilitated by the concerted effort of DNA helicases, a number of which have been shown to interact with RPA following replication fork stalling. Of these, the RecQ family members Werner’s syndrome protein (WRN) and Bloom’s syndrome protein (BLM) appear to play an important role [115-118]. This is highlighted by the cancer-prone syndromes for which they are named, where cells deficient of these proteins display high rates of genetic instability. In functional cells, both BLM and WRN are suggested to suppress excessive homologous recombination by inhibition of RAD51-mediated strand exchange, and the promotion of fork regression [119,120]. *In vitro* data have suggested that this is largely facilitated by the interaction with RPA, which is seen to promote helicase activity [118,121-123]. Interestingly, three OB-folds have recently been identified in the RMI heterodimer, a central component of the Bloom’s helicase complex [124-126]. RMI consists of two proteins, RMI1, which contains 2 OB-folds, and RMI2, which contains 1 OB-fold [124,126]. Here, dimerisation appears to be facilitated by protein-protein interactions between the RMI1 C-terminal OB-fold and the OB-fold of RMI2 [124]. While no detectable DNA-binding activity has been observed for RMI [124], depletion experiments have indicated potential roles in the stabilisation, chromatin localisation, and activity of the BLM complex, including dissolution of Holliday junctions [124-126].

In response to replication fork disruption, RPA also interacts directly with the FANCJ helicase (also known as BRCA1-associated C-terminal helicase; BACH1) [127]. FANCJ is one of 15 known Fanconi anaemia (FA) proteins, the deficiency of which is associated with Fanconi anaemia syndrome [128-130]; like Bloom’s and Werner’s syndrome, FA is characterised by genetic instability [131]. In addition, FANCJ deficiency has been associated with early-onset breast cancer in cells normal for BRCA1 and BRCA2 [132]. Support for a role of FANCJ in the response to replicative stress is provided by sensitisation of cells to hydroxyurea treatment following its depletion [133]. At stalled replication forks, RPA promotes FANCJ processivity and helicase activity [127]. Interestingly, depletion of FANCJ or its binding partner TopBP1 [134,135] decreases RPA-chromatin loading in cells [134]. In addition, FANCJ and TopBP1 depleted cells also show decreased Rad9 and ATR accumulation following hydroxyurea treatment, presumably due to disrupted RPA binding [134]. Recent findings have also demonstrated a direct interaction between the FANCJ C-terminus and BLM helicase following replicative stress [133]. A substantial decrease in BLM protein levels was observed when FANCJ was depleted, which could be rescued by treatment with the proteasome inhibitor MG132, suggesting a potential role for FANCJ in the stabilisation of BLM [133].

RPA has also been seen to localise with SWI/SNF-related, matrix associated, actin-dependent regulator of chromatin, subfamily A-like 1 (SMARCAL1) at stalled replication forks [136-139]. This seems to be largely facilitated by a SMARCAL1 RPA-binding domain similar to that found in TIPIN; this interaction appears necessary for the *in vivo* activity of the protein [137]. Interestingly, SMARCAL1 does not show conventional DNA unwinding helicase activity, but instead functions in the ATPase-mediated re-annealing of unwound ssDNA stretches (known as annealing helicase activity) [140]. In this way, SMARCAL1 appears to function in the regression and remodelling of stalled replication forks [136].

A potential role for hSSB1 in DNA repair at replication forks has also recently been proposed [141]. These data indicate that while *Obfc2b*, the murine homologue of hSSB1, is essential for maintenance of genomic stability, this is through a means other than double-strand DNA break recognition or cell cycle checkpoint activation. Specifically, *Obfc2b* is suggested to function directly in the suppression of replication-associated DNA damage. Additionally, a similar role has been suggested for *Obfc2a* (murine homologue of hSSB2), and indeed a compensatory effect for *Obfc2a* is observed following *Obfc2b* depletion [141,142]. These data potentially represent a role for these homologues in the response to stalled replication forks, and as such, it will be of interest to determine if a similar role exists for hSSB1 and 2.

#### Translesion repair

The choice to repair by HDR or TLS appears largely governed by mono-ubiquitination of the PCNA sliding clamp. Such modification is generally considered to promote recruitment of TLS polymerases to sites of DNA damage [143-145] and indeed, while association between TLS polymerases and non-ubiquitinated PCNA has been demonstrated [146,147], this is with decreased efficiency [148]. Complementary to this event is the poly-ubiquitin-mediated degradation of PCNA during HDR, presumably inhibiting TLS [149]. Recently it has been suggested the switch between PCNA mono- and poly-ubiquitination may be modulated by RPA. This comes from the observation that in cells, RPA interacts directly with RAD18, the E3 ubiquitin ligase which mono-ubiquitinates PCNA following damage [150]. In addition, RPA is seen to interact directly with DNA repair polymerase λ, and to promote correct nucleotide incorporation [151]. Together, these data suggest RPA may play at least two regulatory functions in translesion repair of replicative damage.

### SSBs function in double-strand DNA break repair by homologous recombination

Double-strand DNA breaks (DSBs) are amongst the most cytotoxic DNA damage lesions encountered by the cell, and must be repaired to prevent chromosomal fragmentation. In eukaryotes, one of the major mechanisms through which these lesions are repaired is homologous recombination with a sister chromatid. In this process, DSBs are detected and signalling pathways initiated by the ATM and ATR DNA repair kinases. These signalling cascades serve to recruit repair proteins, including nucleases that resect DNA in a 5^′^-3^′^ direction. This resection generates stretches of ssDNA, which during homologous recombination may invade into a sister chromatid, forming an intermediate structure known as a D loop; this allows the sister chromatid to act as a template for polymerase-mediated extension of the invading strand. In addition, the non-invading strand can be extended using the displaced sister chromatid portion, present in the D loop. Resolution of this mobile junction (the Holliday junction) then yields two intact and identical DNA molecules [152-155].

**Figure 2:**
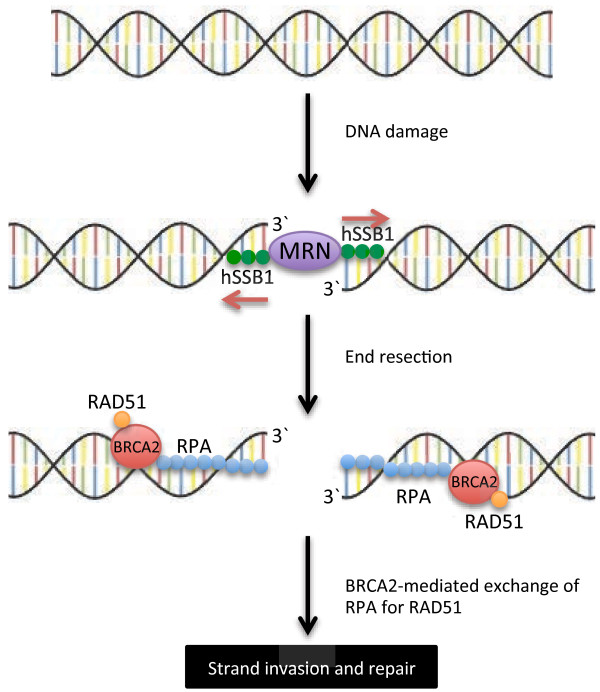
**hSSB1, RPA, and BRCA2 are essential SSBs in the repair of double strand DNA breaks (DSBs) by homologous recombination.** A potential model for human SSBs in the repair of DSBs: The MRN complex is initially recruited to sites of DSBs by hSSB1, allowing for resection of the 5^′^ strand. ssDNA stretches are rapidly bound by RPA, which, following BRCA2-DNA binding, is removed to allow for RAD51 nucleofilament formation. Homologous recombination is then facilitated by RAD51–mediated strand invasion into a sister chromatid. hSSB1 has also been found to interact directly with RAD51 and to facilitate strand invasion *in vitro*, however its precise function at these later stages is unclear.

In cells, the majority of DSBs generated by endogenous events, such as the collapse of replication forks, contain short ssDNA ‘sticky ends’ [156]. When compared with blunt-ended DSBs, the presence of these overhangs is seen to enhance activation of the ATM kinase. ATM is a central DNA repair kinase with downstream substrates involved in cell cycle checkpoint activation (*e.g.,* CHK1, CHK2, p53) and directly in DSB repair (*e.g.,* NBS1, EXO1 and the histone variant H2AX) [157-159]. The recruitment of ATM to sites of DNA damage is largely reliant on DSB recognition by the MRN complex [157,159-161]. MRN is structurally conserved in higher eukaryotes; it contains a MRE11 flexible dimer enfolded by a single NBS1 polypeptide and two RAD50 polypeptides that function to bridge the DNA break [162-165]. Structural analysis has indicated that while the MRE11-RAD50 clamp interacts directly with DNA at DSBs, NBS1 interaction is important in modulating its DNA-binding function [162,166]. Furthermore, crystallography data of the *Saccharomyces pombe* complex has indicated the NBS1 N-terminus is orientated in such a way as to interact directly with DSB foci proteins located near the MRE11-RAD50 binding cleft [162]. Of these interacting proteins, hSSB1 is seen to have a central role in DSB repair by homologous recombination [7,34]. Here, hSSB1 rapidly interacts with ssDNA overhangs at sites of DSBs, where it functions as an initial recogniser of these breaks [34,37] (Figure 2). This has been supported by *in vitro* data, where hSSB1 was found to bind duplex DNA with 6 bp ssDNA overhangs, as well as, to a lesser degree, natural breathing sites of a short 33 bp dsDNA oligo [34]. Additionally, laser micro-irradiation of cells has demonstrated hSSB1 binding at sites of DSBs within 10 seconds of DNA damage in all interphase cells [37]. hSSB1 recruitment to DSBs is also independent of CtIP, MDC1 or MRN [37]. Furthermore, MRN recruitment at sites of DSBs is substantially inhibited in cells deficient of hSSB1 [37]. As the hSSB1 C-terminus interacts directly with NBS1, this suggests that hSSB1 may participate in the immediate recruitment of the MRN complex to DSB foci [34]. Further *in vitro* data has supported this, demonstrating that MRN nuclease activity, which is known to be weak, is substantially stimulated in the presence of hSSB1 [34].

Both hSSB1 and 2 have recently been shown to form separate heterotrimeric protein complexes (sensor of single-stranded DNA 1 and 2; SOSS1 and 2) with the integrator complex subunit 3 (INTS3) and hSSB-interacting protein 1 (hSSBIP1) [35,167,168]. These complexes are both structurally and functionally unlike the RPA heterotrimer, and neither INTS3 nor hSSBIP1 have yet been shown to have ssDNA-binding affinity. In addition, while hSSB1 and hSSBIP1 co-localisation has been observed at sites of DNA damage [167], inconsistent findings have been reported regarding the localisation of INTS3 and MRN at DNA damage sites. Interestingly, the addition of SOSS1 components has however recently been shown to promote exonuclease 1 (EXO1) DSB resection *in vitro*[36], although it remains unclear whether this is due to activity of the SOSS1 complex, or dissociated hSSB1. Furthermore, while reduced clonogenic survival of cells depleted of SOSS1 and SOSS2 components and exposed to DNA damaging agents has been observed, INTS3 and hSSBIP1 depletion caused only a slight reduction in homologous recombination-dependent DSB repair, when compared to depletion of hSSB1 or 2 [167]. As there is compounding evidence to suggest INTS3 associates at DNA damage sites at later time points (4–6 hr following damage) [35,169], one explanation for these findings may be that the SOSS1 complex functions in DSB repair only during the later phases. Alternatively, as INTS3 depletion has been shown to destabilise hSSB1 and 2 [167,168], as well as to decrease the transcriptional rate of hSSB1 [35], this may suggest INTS3 modulates DSB repair through the regulation of hSSB1 and 2, as opposed to a direct means. Interestingly, while most known SSBs bind ssDNA with four OB-folds, recent data has indicated hSSB1 may exist as part of the SOSS1 complex in a monomeric form [36]. Furthermore, while the complex contains a single hSSB1 OB-fold, this domain is also known to interact with INTS3 [35,169], and so may indeed not be available for DNA binding.

An essential role of the MRN complex is the resection of DSB 5^′^ strands to expose stretches of ssDNA for RAD51-mediated strand invasion. While the MRE11 component has both exo- and endo-nuclease activity, only the endonuclease function seems to be required for DSB processing [170,171]. For this, MRE11 nicks the target strand up to 300 bases from the DSB site, and resects the strand in the 3^′^-5^′^ direction. This is complemented by the 5^′^-3^′^ exonuclease activity of EXO1, which concurrently digests the target strand away from the DSB site [171]. The activation of MRN nuclease activity is stimulated by interaction with C-terminal binding protein interacting protein (CtIP) [172]. Interestingly, MRN and CtIP have been found to interact directly with BRCA1, an association necessary for efficient end resection and loading of RPA onto ssDNA [173-175]. The association of RPA with ssDNA is likely important for the protection of the otherwise exposed ssDNA strand, preventing it from damage and digestion, or the formation of disruptive secondary structure [176].

RPA ssDNA-binding is also necessary for the recruitment of downstream proteins involved in active DNA repair and checkpoint signalling. Of central importance is the interaction with the RAD51 recombinase, a protein which, following RPA displacement, rapidly coats ssDNA strands [177,178]. Although it remains unclear exactly how this interchange occurs, several interactions appear necessary for both the removal of RPA, and the loading of RAD51. Of these, the direct interaction of RPA and RAD51 appears to play a central role. Here, the A-OB-fold of ssDNA-bound RPA interacts directly with the RAD51 N-terminus, potentially representing a means of competitive ssDNA-binding [179]. One mechanism through which this has been suggested to occur is the capture of transiently bound RPA, presumably preventing its re-association with the ssDNA strand [179,180]. In addition, the RPA-Rad51 exchange seems to be promoted by Rad52, a protein shown to bind the RPA1 and 2 subunits, and to stimulate their displacement [181-183]. This interaction seems to be promoted by phosphorylation of RPA1, an event that also stimulates ssDNA exchange [184].

The displacement of RPA, which is essential for completion of homologous recombination, also appears to be promoted by the OB-fold containing protein, BRAC2 [185]. Structural analysis of this protein has indicated the presence of a DNA-binding region composed of a helical domain, followed by three OB-folds [186]. Interestingly, the central OB-fold also contains a protruding structure composed of five α-helices, three of which form a helix-turn-helix bundle which sits atop the remaining two anti-parallel helices; this structure is referred to as a ‘tower domain’. While the helical domains and three OB-folds may bind ssDNA, the tower domain seems to bind dsDNA [186]. This dual-binding ability presumably allows BRCA2 to interact efficiently with both ssDNA and dsDNA found at the ends of a resected DSB, as has been observed for the Brh2 nuclease, the *Ustilago maydis* fungal homologue of BRCA2 [187]. In this manner, it seems likely the DNA binding activity of BRCA2 may be involved in modulating RPA-ssDNA-binding, potentially displacing RPA through competitive ssDNA association. In addition to binding DNA, the first and second OB-folds and the tower domain have also been found to interact with deleted in split-hand/split-foot syndrome 1 (DSS1), a protein associated with BRAC2 stability and modulation of DNA binding activity [186,188,189]. Although a direct interaction between the RPA complex and the GST-tagged N-terminus of BRCA2 has been suggested [190], this has not been supported by more recent studies using full-length BRCA2 [191].

BRCA2 also interacts directly with RAD51 through a series of eight 35 amino acid repeat motifs, known as the BRC repeats [192-195]. Remarkably, the crystal structure of BRC4 bound to RAD51 identified a conserved BRC fingerprint that mimics the RAD51 homodimer interface; this may potentially support stabilisation of the RAD51 monomeric form [196]. Such stabilisation presumably represents a rationale for interaction of the BRC repeats in RAD51 loading [197,198]. This has been supported by *in vitro* data, where BRCA2 was shown to increase RAD51-mediated DNA strand exchange of an RPA-coated ssDNA construct [191]. Additionally, a 7-fold increase in RAD51 binding to RPA-coated ssDNA has been observed in the presence of BRCA2, suggesting a role for BRCA2 in RAD51 nucleofilament formation [199]. Recently the BRC repeats have been further classified into two distinct classes, distinguished by their differing RAD51-binding affinities; while the first series (BRC1-4) bind the free protein with high affinity, the second (BRC5-8) strongly interact only when RAD51 is in a filamentous ssDNA-bound form [200]. The existence of these two classes supports the notion that BRCA2 functions in both loading and stabilisation of RAD51 nucleofilaments.

hSSB1 and RAD51 have also been found to co-localise following ionising radiation exposure, which, on the basis of immunoprecipitation data, could be facilitated through a direct interaction [7]. This suggests that hSSB1 may potentially share an overlapping role with RPA in the modulation of Rad51-mediated strand invasion. Such a suggestion is consistent with *in vitro* data, where hSSB1 was able to stimulate Rad51-mediated strand exchange to a similar degree as RPA [7]. Further, depletion of the hSSB1 binding partner, INTS3, was shown to suppress RAD51 foci formation, as well as the recruitment of topoisomerase binding protein 1 (TopBP1) and BRCA1 to sites of DNA damage [169]. Interestingly however, using high-resolution microscopy, RPA and hSSB1 were not shown to directly localise at radiation-induced foci, but to form proximal centres [7]. This may suggest that while functional overlap between these proteins exists, their individual roles at each DSB site may be independent, representing different stages of processivity.

### Nucleotide excision repair requires the action of SSBs

Nucleotide excision repair is an important pathway in the maintenance of genomic stability, as it represents an essential mechanism through which the cell is able to remove a large number of structurally varied adducts. These adducts may arise through either endogenous or exogenous means and are recognised as a result of the ensuing disruption to base pairing and helical distortion, as opposed to their precise chemical nature [153]. However, adducts caused by ultraviolet (UV) light-induced damage, such as cyclobutane pyrimidine dimers (CPDs) and 6–4 photoproducts, do represent primary targets. This is demonstrated by the conditions Xeroderma pigmentosum (XP), Cockayne syndrome (CS) and Trichothiodystrophy (TTD); these conditions are all associated with diminished NER and severe photosensitivity [201]. In functional cells, NER may occur through two related pathways: global genomic NER (GG-NER), which removes lesions non-specifically throughout the genome, and transcription-coupled NER (TC-NER), which removes lesions from actively transcribed genes.

RPA has a well-established role in NER, where it functions to both stabilise exposed ssDNA and to recruit repair proteins at these sites [202,203]. Of these, the XP proteins (XPA – XPG) play a direct function both in the recognition and excision of damaged nucleotides [204]. In the earliest stages of NER, damage is recognised by a complex of the XPC protein and hHR23B [205]. This allows unwinding of the lesion, a function further facilitated by the helicase activity of the TFIIH transcription factor [206]. Additionally, this process is stimulated by XPA, which recognises damaged DNA and seems to function in the recruitment of TFIIH [207,208]. Here, XPA binding of damaged DNA is promoted through the direct interaction with RPA [204,209,210]; this interaction is facilitated both by the RPA1 and 2 subunits [211,212]. Through promotion of TFIIH helicase activity (facilitated by the XPB and XPD components), RPA-XPA expands the damage region [213,214]. RPA2 also interacts directly with the XPF-ERCC1 exonuclease, while RPA1 interacts with XPG [31,215-218]. As a result of the differential interaction regions for these proteins, and the binding polarity of RPA, RPA can correctly position XPF-ERCC1 and XPG at sites of damage; XPG is recruited 3^′^ to the lesion, while XPF-ERCC1 binds on the 5^′^ side [31,215,217]. In addition, by binding the undamaged DNA strand, RPA is able to direct ERCC1-XPF nuclease activity to the site of damage on the opposing strand [31].

In response to UV treatment, RPA2 phosphorylation is mediated through the combined efforts of the ATR and DNA-PK kinases, leading to replication arrest [69,71,72,78,97,219]. Interestingly, hSSB1 is also stabilised following exposure to UV, and is localised to sites of the resulting DNA damage [7]. It will therefore be of interest to determine whether hSSB1 plays a role in NER.

### SSBs are necessary for cell cycle checkpoint activation following DNA damage

An essential step to prevent the propagation of damaged DNA, either by its replication or division amongst daughter cells, is the damage-induced suppression of cell cycle progression. This is achieved through the activation of cell cycle checkpoints, a cascade of signalling events resulting in the inhibition of cyclin/CDK-mediated checkpoint progression [220,221]. In mammalian cells, initiation of checkpoint signalling is largely facilitated by two members of the PI3 kinase-related kinases family, ATM and ATR [222-224]. For both ATM and ATR, activation results in the initiation of downstream signalling, facilitating activation of effector kinases, namely Chk1 and Chk2 [225]. Although Chk1 activation is predominantly mediated by ATR, and Chk2 activation predominantly by ATM, considerable crossover is also apparent between these pathways [225-230]. In addition, a temporal contrast exists in the primary initiation of ATM and ATR signalling; while ATM is activated prior to DSB resection as a result of MRN recruitment [221], ATR activity is stimulated by RPA-coating of ssDNA strands [89,231,232].

**Figure 3:**
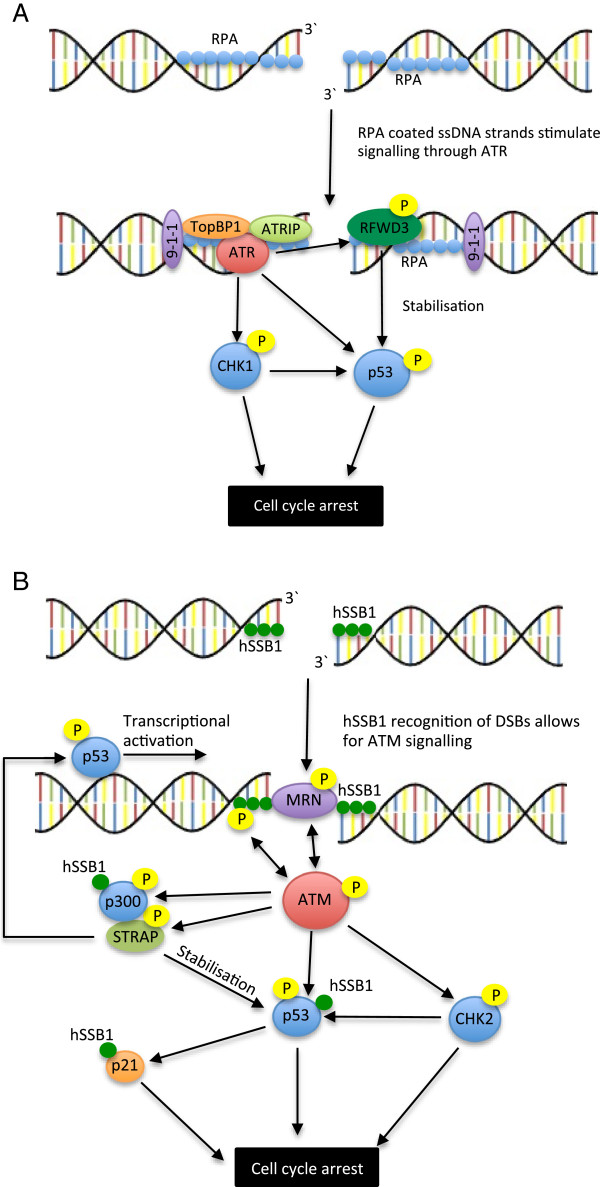
**SSBs function in cell cycle checkpoint activation.** (**A**) Schematic of the potential role of RPA in ATR signalling: RPA coated ssDNA stimulates ATR signalling both through direct interaction with its binding partner ATRIP, as well as through the 9-1-1 complex. Activated ATR phosphorylates various cell cycle checkpoint proteins, including p53 and CHK1, allowing for cell cycle arrest. RPA also interacts with RFWD3, an E3 ubiquitin ligase involved in p53 stabilisation. (**B**) Schematic of the potential role of hSSB1 and Strap in ATM signalling: hSSB1 activation allows for the recruitment of the MRN complex, which activates the ATM kinase. ATM phosphorylation of hSSB1 then facilitates a positive feedback loop, which further activates ATM, as well as many downstream ATM targets. hSSB1 also interacts with and stabilises p53 and p21 (an effector of p53 signalling), as well as the p53-interacting acetyltransferase p300. Strap, a phosphorylation substrate of ATM, interacts with and stabilises p53, and additionally directs it to transcriptional targets.

ssDNA-bound RPA partially activates ATR through a direct interaction between the RPA1 N-terminus and the ATR-interacting protein (ATRIP) [89,161,232] (Figure 3A). This is supported by suppressed phosphorylation of the CHK1 and Rad17 ATR substrates following RPA1 depletion [89]. Disruption of the RPA-ATRIP interaction is however not sufficient to eliminate localisation of the ATR-ATRIP complex to sites of DNA damage [232], and full RPA-mediated activation requires an additional interaction with the checkpoint complex Rad9/Rad1/Hus1 (9-1-1)/ Rad17-Rfc2-5 [89,233,234]. Here, the 9-1-1 component forms a PCNA-like sliding clamp [235], which seems to interact directly with the RPA1 N-terminal OB-fold [94], as well as with the topoisomerase-binding protein 1 (TopBP1) [224,236,237]. In turn, recruitment and activation of TopBPI is seen to stimulate the kinase activity of ATR, amplifying downstream signalling [238].

Recent data has suggested that RPA also mediates p53 activity by interaction with the RING finger and WD repeat domain 3 (RFWD3) E3 ubiquitin ligase [239,240]. RFWD3 has been identified through proteomic screening as a substrate of ATR kinase [241-243], and is important for CHK1 activation [239]. In response to replication-stress and DNA damage, RFWD3 is rapidly recruited to RPA coated ssDNA stretches [239,240,244]. Here it interacts directly with murine double minute 2 (Mdm2), the major E3 ubiquitin ligase involved in p53 poly-ubiquitination [244]. While suppression of p53 poly-ubiquitination immediately following damage appears to be facilitated through various post-translational means [245], competitive ubiquitination by RFWD3 seems to present a method of stabilisation during the later phase (after 2.5 hours) [244]. This may occur through the generation of smaller non-proteasome-targeting ubiquitin chains. In addition to this role, RFWD3 may also have a more direct role in DSB repair, as suggested by disrupted RPA phosphorylation following DNA damage in RFWD3 depleted cells [240]. As yet however, the mechanism through which this occurs remains unclear.

Activation of the ATM kinase is one of the most immediate consequences of MRN recruitment to DSB foci. In addition to a large number of other substrates, this activation allows for the ATM-mediated phosphorylation of hSSB1, facilitating a positive feedback loop that amplifies ATM signalling (Figure 3B). This was demonstrated by mutation of the ATM-mediated hSSB1 phosphorylation site (T117), which led to suppression of ATM auto-phosphorylation [7]. In addition, depletion of hSSB1 from cells demonstrated defective ATM-checkpoint signalling, including loss of G1/S and G2/M checkpoint activation, as well as suppressed NBS1, p53, CHK1 and CHK2 phosphorylation [7]. Interestingly, despite this central role for hSSB1, hSSB2 has been reported as dispensable for ATM activation and checkpoint arrest [1].

hSSB1 has also been seen to interact directly with the cyclin-dependent kinase inhibitor p21 [246], an important effector of p53-mediated G1-S and G2-M checkpoint arrest [247-251]. This is achieved through the inhibition of cyclin E/CDK2 and cyclin B/CDK2 activity, arresting cell cycle progression. In response to DNA damage, hSSB1 was suggested to bind p21 [246], where it inhibits the ubiquitin-mediated degradation associated with the labile protein [252-254]. More recent data has indicated that hSSB1 may also function to promote p53 stabilisation through a direct interaction [255]. Additionally, hSSB1 depletion was found to suppress p300-mediated acetylation of p53 [255], an important modification associated with p53 transcriptional activity. This was supported by the decreased expression of p21 and SULF2, two p53 transcriptional targets [247,256], following hSSB1 depletion [255]. Together, these data suggest a further upstream function of hSSB1 in the p53 pathway; in addition to amplification of ATM signalling, hSSB1 may regulate the G1/S and G2/M transitions.

Recently, a novel OB-fold was reported in Strap, an important cofactor of p53 [257,258]. In response to DNA damage, ATM-mediated phosphorylation of Strap allows for nuclear accumulation of the protein, while protein stabilisation is achieved following phosphorylation by CHK2 [259,260]. This is supported by the observation that in ataxia telangiectasia cells, or cells expressing Strap mutated at the S203 ATM phosphorylation site, Strap is restricted to the cytoplasm [260]. Following damage, localised Strap interacts with other components of the p300 coactivator complex, including p300, the junction mediating and regulatory (JMY) protein, and protein arginine methyltransferase 5 (PRMT5) [258,261-263]. Such protein interactions are facilitated by both the Strap N-terminus, which contains six tetratricopeptide repeat (TPR) motifs [258], as well as the C-terminus, which largely consists of the OB-fold [257]. Here, JMY is bound by two N-terminal domains [258], identified as TRP motifs 1–3, and 4–5, while TRP6 and the OB-fold interact directly with p300 [257]. As part of the p300 complex, Strap has been shown to promote p53 activity both by promoting stabilisation of p53 through suppression of MDM2-mediated poly-ubiquitination, as well as by stimulating p53 transcription modulating activation [258]. In agreement with the later, *in vitro* data has indicated the Strap OB-fold is able to bind ssDNA and dsDNA, while chromatin immunoprecipitation has demonstrated localisation of Strap, as well as the TRP and OB-fold domains, at p53 target genes [257].

### ssDNA at telomere ends are bound by SSBs

The replication of chromosome ends presents a unique challenge in eukaryotic cells. This arises from the strict requirement of a primed template from which 5^′^-3^′^ extension can occur, a constraint preventing the replication of chromosome 3^′^ terminal regions. To overcome this ‘end-replication problem’, eukaryotic chromosome ends are comprised of protein-nucleic acid structures known as telomeres. These structures contain telomeric DNA arranged in a series of repeats, the sequence of which varies between organisms. In humans, telomeric DNA is composed of precise hexanucleotide (CCCTAA/TTAGGG) repeats constituting 2–50 kb of code (3,000-80,000 repeats) [21,264,265]. While telomere repeats are re-synthesised in germ and embryonic cells by the enzyme telomerase, in somatic cells, shortening of chromosomes to a critical length causes the induction of a senescence phase [264].

An additional consequence of incomplete lagging strand replication is the generation of 3^′^ ssDNA overhangs on each chromosome end. The precise length of these overhangs is fundamentally determined by the positioning of the final RNA primer, generally ranging in human cells from 30–40 nt for leading strand, and 80–120 nt for lagging strand, daughter chromosomes [265,266]. The presence of these overhangs offers an additional challenge for eukaryotic cells, as to prevent their degradation or deleterious recognition as a site of DNA damage, they must be in some way sequestered [21]. One mechanism through which this occurs is the formation of a T-loop structures, in which the 3^′^ overhangs invade an upstream telomere duplex region, and by virtue of the telomere hexanucleotide repeats, form stable interactions at the base of the T-loop [267,268]. However, while this shields the 3^′^ termini, ssDNA is still exposed in the displaced section of the telomere duplex (the D-loop). To protect these regions, telomeric ssDNA is protein bound, forming a telomere ‘caps’ [24].

An important component of the telomere cap is the shelterin/telosome complex, a protein complex composed of six subunits: TRF1, TRF2, TIN2, TERF2IP, TPP1, and POT1 [269]. Of these, protection of telomeres 1 (POT1) and TPP1 (also known as POT1-interacting protein; PIP1), form a DNA-binding heterodimer that interacts directly with teleromic ssDNA [270,271]. Although OB-folds have been detected in both proteins, DNA-binding is predominantly facilitated by POT1, which interacts with strong specificity to 5^′^-(T)TAGGGTTAG-3^′^ sequences in the T- and D- loops [272-274]. Crystallography data has indicated that POT1 contains tandem OB-folds, both of which bind ssDNA. This is facilitated by the special arrangement of the OB-folds, where the binding grooves of each domain form a continuous channel, with both OB-folds arranged in-line [273]. In addition, the second of these domains is somewhat modified from the archetypal OB-fold, containing a 31-residue insertion between the first and second β-barrels, similar to that observed in the CTC1 DNA binding domain [275]. While suggested not to bind ssDNA directly, a third POT1 OB-fold has also been suggested in the TPP1 interaction region [271]. In addition, TPP1 also contains a putative OB-fold [270], although it does not appear to bind ssDNA directly, and instead TPP1 localisation at telomeric DNA is mediated by POT1 [270,271]. A protein-interaction function has however recently been described for the TPP1 OB-fold, where the domain was observed to bind and recruit the telomerase reverse transcriptase (TERT) [276-278]. Interestingly, this is in addition to the observation that POT1-TPP1 binding delays primer dissociation [279], suggesting at least two possible mechanisms through which the dimer may function in telomere processivity. Contrasting this idea however is the observation that, *in vitro*, POT1 binding of 3^′^ terminal ends sterically inhibits telomerase access [273]. Furthermore, deletion of the POT1 N-terminal OB-fold resulted in an increase in telomere length *in vivo*[280]. Together, these seemingly conflicting roles may indicate the POT1-TPP1 heterodimer is involved both in the stimulation and suppression of telomerase activity, however as yet the coordination of these activities remains unclear.

In addition to binding DNA by virtue of the POT1-TPP1 heterodimer, the telomeric repeat-binding factors 1 and 2 (TRF1 and 2), as well as TRF2 interacting protein (TERF2IP, also known as RAP1), also allow the shelterin complex to bind dsDNA at sites adjacent to the T-loop [269,281]. As with POT1, DNA-binding of TRF1 and 2 is highly sequence specific, with both proteins recognising telomeric duplex DNA of sequence 5^′^-GTTAGGGTTAGGG-3^′^[282-284]. Such sequence-specificity is however not evident for TERF2IP, where *in vitro* data has demonstrated similar binding of the protein with telomeric and non-telomeric DNA [281]. Even so, TERF2IP recruitment remains sequence-specific *in vivo*, based on the requirement of TERF2 interaction for efficient DNA binding [285]. Another important factor of teleromic DNA protection is stabilisation of the shelterin complex, a process facilitated by the scaffolding protein TIN2, which couples TRF1 with TRF2, as well as linking TRF1 to TPP1 [279,286,287]. Additionally, this also represents a mechanism through which TRF1 and 2 are able to recruit POT1 by indirect interaction with TPP1 [288,289].

A central function of the shelterin complex is to prevent induction of a DSB response against exposed telomeric DNA. Recently this has been demonstrated by double knockout of TRF1 and 2 in mouse embryonic fibroblasts, leading to generation of shelterin-free telomeres. Here, both ATM and ATR responses were elicited against the exposed chromosome ends, as indicated by the accumulation of 53BP1 foci, CHK1 and 2 phosphorylation, and increased telomere fusion events [290]. These data were consistent with previous observations in senescent cells [291,292], as well as cells where TRF2 and/or POT1 was depleted [293-296]. Interestingly, TRF2 and POT1 seem to play independent roles in the suppression of DSB signalling; while ATM suppression seems to occur through TRF2, POT1 is involved in the prevention of ATR activation [293]. Here, POT1 suppression of ATR signalling was suggested to be through the steric inhibition of RPA localisation, a process described in previous sections as essential for such signalling. Interestingly however, RPA is known to bind telomeres during S-phase where it is suggested to promote telomerase activity [297,298]. Although POT1 is unable to displace RPA on its own, the switch from RPA to POT1 binding seems to be facilitated outside of S-phase by the heterogenous nuclear ribonucleoprotein A1 (hnRNPA1) [299].

An additional protein complex central for the capping of telomeres is composed of the proteins CTC1 (CDC13), STN1 and TEN1 [24,300-302]. Together, this complex (CST) constitutes a ssDNA-binding unit which interacts independently of POT1 [301]. Furthermore, as each of these subunits contain putative OB-fold domains, structural and functional similarities have been drawn with the RPA heterotrimer [302,303]. An exception to this is the presence of an additional C-terminal winged helix-turn helix (wHTH) motif on the STN1 subunit, which confers a telomere-specific binding function [24]. CTC1 appears to contain 3 OB-folds, in contrast to the 4 of RPA1, two of which have been further confirmed by structural analysis [275,304-306]; here, ssDNA-binding is largely facilitated by the CTC1 C-terminal OB-fold [307,308]. Currently the end-protection role of CST remains unclear, and indeed while some authors have reported de-protection of telomeres following CST component depletion [301,309], others have suggested only a minimal effect [302,310]. Furthermore, while it seems uncontested that CST functions in telomere length control, both telomere lengthening [302], and shortening [310] has been reported in cells deficient of CST. As the N-terminal OB-fold of CTC1 is known to interact with telomerase [304,308], these length control functions are likely due to regulation of telomerase activity. As yet the coordination of this function, as with the POT1-TPP1 dimer, remains unclear.

## Summary

SSBs from the OB domain family play an essential role in the maintenance of genome stability, functioning in DNA replication, the repair of damaged DNA, the activation of cell cycle checkpoints, and in telomere maintenance. The importance of SSBs in these processes is highlighted by their ubiquitous nature in all kingdoms of life [1]. Here, in addition to genome stability maintenance, SSBs function in all known processes involving the exposure of ssDNA, such as transcriptional activation [311]. In humans, RPA has long been known to play an important role in the processing of ssDNA, however the recent identification of hSSB1 and 2 has raised several questions regarding the coordination of these processes. Additionally, the diversity of OB-fold primary sequences has made it difficult to detect these domains by non-biophysical means, allowing for the continued identification of OB-folds in previously identified proteins. This is highlighted by the recent identification of Strap and the RMI dimer, and suggests that further SSBs are likely to be identified.

## Competing interests

The authors declare that they have no competing interests.

## Authors’ contributions

NWA, EB, and LC contributed significant intellectual input to both writing and editing. DJR and KJO contributed significant intellectual input to content, structure and editing. All authors read and approved the final manuscript.
